# The role of maternal homocysteine concentration in placenta-mediated complications: findings from the Ottawa and Kingston birth cohort

**DOI:** 10.1186/s12884-019-2219-5

**Published:** 2019-02-19

**Authors:** Shazia H. Chaudhry, Monica Taljaard, Amanda J. MacFarlane, Laura M. Gaudet, Graeme N. Smith, Marc Rodger, Ruth Rennicks White, Mark C. Walker, Shi Wu Wen

**Affiliations:** 1 The Ottawa Hospital Research Institute, Ottawa, Ontario Canada; 2 School of Epidemiology and Public Health, University of Ottawa, Ottawa, Ontario Canada; 3 Nutrition Research Division, Health Canada, Ottawa, Ontario Canada; 4 Department of Biochemistry, Microbiology and Immunology, University of Ottawa, Ottawa, Ontario Canada; 5 Department of Obstetrics & Gynaecology, Division of Maternal-Fetal Medicine, Queen’s University, Kington, Ontario Canada; 6 Kingston General Hospital Research Institute, Kington, Ontario Canada

**Keywords:** Homocysteine, Hyperhomocysteinemia, Pregnancy complication, Placenta, Preeclampsia, Small for gestational age, Placental abruption, Pregnancy loss

## Abstract

**Background:**

Homocysteine is an intermediate metabolite implicated in the risk of placenta-mediated complications, including preeclampsia, placental abruption, fetal growth restriction, and pregnancy loss. Large cohort and case-control studies have reported inconsistent associations between homocysteine and these complications. The purpose of this study was to investigate whether elevated maternal plasma homocysteine concentration in the early to mid-second trimester is associated with an increased risk of placenta-mediated complications. We examined the following potential moderating factors that may explain discrepancies among previous studies: high-risk pregnancy and the MTHFR 677C>T polymorphism.

**Methods:**

We analyzed data from participants recruited to the Ottawa and Kingston (OaK) Birth Cohort from 2002 to 2009 in Ottawa and Kingston, Canada. The primary outcome was a composite of any placenta-mediated complication, defined as a composite of small for gestational age (SGA) infant, preeclampsia, placental abruption, and pregnancy loss. Secondary outcomes were, individually: SGA infant, preeclampsia, placental abruption, and pregnancy loss. We conducted multivariable logistic regression analyses with homocysteine as the primary continuous exposure, adjusting for gestational age at the time of bloodwork and explanatory maternal characteristics. The functional form, i.e., the shape of the homocysteine association with the outcome was examined using restricted cubic splines and information criteria (Akaike’s/Bayesian Information Criterion statistics). Missing data were handled with multiple imputation.

**Results:**

7587 cohort participants were included in the study. Maternal plasma homocysteine concentration was significantly associated (linearly) with an increased risk of both the composite outcome of any placenta-mediated complication (*p* = 0.0007), SGA (*p* = 0.0010), severe SGA, and marginally with severe preeclampsia, but not preeclampsia, placental abruption and pregnancy loss. An increase in homocysteine concentration significantly increased the odds of any placenta-mediated complication (odds ratio (OR) for a 5 μmol/L increase: 1.63, 95% Confidence Interval (CI) 1.23–2.16) and SGA (OR 1.76, 95% CI 1.25–2.46). Subgroup analyses indicated some potential for modifying effects of the MTHFR 677C>T genotype and high-risk pregnancy, although the interaction was not statistically significant (high-risk subgroup OR 2.37, 95% CI 1.24–4.53, *p*-value for interaction =0.14).

**Conclusions:**

Our results suggest an independent effect of early to mid-pregnancy elevated maternal homocysteine on placenta-mediated pregnancy complications.

**Electronic supplementary material:**

The online version of this article (10.1186/s12884-019-2219-5) contains supplementary material, which is available to authorized users.

## Background

Maternal plasma homocysteine concentration is proposed to be associated with certain pregnancy complications [1]. Based on evidence supporting the role of homocysteine in endothelial dysfunction and as a risk factor for cardiovascular disease, elevated maternal homocysteine is hypothesized to play a role in placenta-mediated pregnancy complications (PMCs), including preeclampsia, placental abruption, intrauterine growth restriction (IUGR), and pregnancy loss [2–5]. All have been linked to abnormal placental vasculature, share a common placental pathophysiology, and have an increased risk to reoccur [6, 7].

Within the 1-carbon metabolic cycle, homocysteine is an intermediate metabolite formed in the methionine cycle. Homocysteine can be transmethylated to form methionine, which in turn is converted to S-adenosylmethionine, the main cellular methyl donor from which methyl groups can be transferred to multiple recipient molecules, including DNA and histones. The complex cycle involves key co-enzymes and co-factors including vitamins B_9_ (folate), B_6_ and B_12_. Polymorphisms in genes related to 1-carbon metabolism, as well as various modifiable lifestyle and behavioural factors are associated with elevated homocysteine [8, 9].

Studies have reported inconsistent associations between maternal homocysteine, measured at different time points in pregnancy and placenta-mediated complications [10, 11]; even among larger cohort and case-control studies measuring homocysteine from early pregnancy, the associations are inconsistent [12–18]. The discrepancies could be due to moderating factors like high-risk pregnancy and differences in population frequencies of the MTHFR 677C>T polymorphism that can lead to moderately elevated homocysteine [19, 20]. Discrepancies could also arise from different percentile cut-offs used to define elevated homocysteine [13, 14, 21].

The purpose of this study was to investigate whether elevated maternal plasma homocysteine concentration measured in the early to mid-second trimester of pregnancy is associated with an increased risk of PMCs. Our analytic approach sought to explore potential non-linear effects of homocysteine concentration and other continuous factors so as to retain as much information about the association, which can otherwise be lost through categorizing continuous variables [22]. We also sought to determine whether the association is modified by the MTHFR 677C>T genotype and by high-risk pregnancy.

## Methods

### Study design

Women attending prenatal appointments and planning to deliver at The Ottawa Hospital, the Ottawa region, or the Kingston General Hospital were recruited to the Ottawa and Kingston (OaK) Birth Cohort from 2002 to 2009. Participants were included if between 12 and 20 weeks gestation of a viable singleton or twin pregnancy. For the present study, participants were excluded from the analytic data set if non-singleton, recruited before 12 or after 20 weeks gestation, if they withdrew, were lost to follow-up, or if the pregnancy was terminated.

Details of the cohort study have been previously reported [23]. Briefly, the baseline survey and post-partum follow-up consisted of an interviewer-administered questionnaire and hospital record abstraction. Maternal blood samples were collected at baseline and laboratory personnel were blind to outcomes. Blood samples for homocysteine and MTHFR were collected in K_2_EDTA Vacutainer tubes (Becton Dickinson, Lincoln Park, NJ) and for serum folate in serum separator tubes (Becton Dickinson). Samples for plasma were immediately placed on ice and within 30 min centrifuged in 4 °C at 3000×*g* for 10 min, then aliquoted and stored at − 20 °C. Plasma homocysteine (μmol/L) was measured on the Abbott AxSYM II Immunoassay System (Abbott Laboratories, Abbott Park, IL) using fluorescence polarization immunoassay. Blood samples for serum were centrifuged at 3000×*g* for 10 min, then aliquoted and stored at − 20 °C. Serum folate (nmol/L) was measured using the Beckman Coulter Access 2 and Unicel DxI 800 immunoassay analyzers using manufacturer’s reagents (Beckman Coulter, Brea CA). Homocysteine and folate were measured within one month in batches.

### Outcomes

The primary outcome was the composite of placenta-mediated complications (PMCs): small for gestational age (SGA), preeclampsia, placental abruption, and pregnancy loss. The secondary outcomes were individual components of the composite. Additionally we investigated severe SGA <5th percentile and severe preeclampsia.

A small for gestational age infant had a birth weight less than the 10th or 5th percentile of sex and gestational age-adjusted population standards [24]. Pregnancy loss was intrauterine death before 20 weeks or stillbirth. Preeclampsia was new onset hypertension with proteinuria. Hypertension was a diastolic blood pressure reading greater than or equal to 90 mm Hg and proteinuria of 2+ on a dipstick or proteinuria greater than 300 mg in a 24-h urine collection measured on two separate occasions of at least 6 h apart. Preeclampsia with delivery prior to 35 weeks gestation was considered severe [25]. Placental abruption was antepartum bleeding with objective evidence either on ultrasound or inspection of the placenta at birth or a pathologic examination of a retro-placental clot [26]. A committee of medical experts blind to participant exposure status independently examined information abstracted from medical records to adjudicate preeclampsia and placental abruption.

### Potential risk factors

Potential confounders, risk factors, and interactions were specified prior to multivariable modeling using knowledge of the subject matter, our analyses of homocysteine determinants in OaK participants (unpublished), and previous work investigating PMCs [12–14, 21, 27]. We considered the following factors: maternal age, race, education, income, nulliparity, smoking, diabetes, use of hormonal birth control prior to conception, chronic hypertension, history of PMCs, folic acid supplementation, and serum folate. Gestational age at recruitment was abstracted from hospital records.

#### Statistical analyses

All statistical analyses were performed in SAS® version 9.4 and RStudio version 0.99.892, R version 3.2.3 [28] and statistical significance was assessed at the 5% level. A variable clustering algorithm was used to examine multicollinearity among independent variables. Missing data patterns were visualized and multiple imputation was performed using multivariate imputation by chained equations [29, 30]. This approach uses sequential regression imputation to create multiple “complete” datasets, a procedure that is flexible and generates multiple predictions for each missing value as a function of all observed data (including auxiliary variables), taking into account the type of variable (i.e., continuous, binary, categorical, ordinal). The number of imputations was set to 10 which is considered an adequate number of imputations [30–32].

### Regression analyses

We conducted multivariable logistic regression analyses to examine the association of plasma homocysteine concentration (as the primary exposure of interest) with PMCs, while adjusting for the identified potential confounders and risk factors. We did not transform homocysteine because log-transformation did not substantially normalize the distribution (Additional file 1: Figure A). Because serum folate is intermediate with respect to folic acid supplementation, analyses were conducted with and without serum folate to note any changes in effect estimates for folic acid supplementation. Changes were also noted for outliers in homocysteine concentration.

Multivariable model building was conducted as follows [22]. An initial model was fit including main effects for all independent variables. Continuous variables, i.e., homocysteine, gestational age, maternal age, BMI, and serum folate were modeled using restricted cubic splines with five knots set at the 5th, 27.5th, 50th, 72.5th, and 95th quantiles. Next, a plot of partial associations referred to as an ANOVA plot, corrected for the number of degrees of freedom, was generated to visualize strong and weak partial associations. The strengths of association informed a decision of how many degrees of freedom to allocate to each variable: strong associations were modeled with greater complexity than weak associations. For example, weaker partial associations in continuous variables were reduced to fewer knots or a linear term, while categorical and ordinal variables were collapsed. Akaike’s Information Criterion (AIC) and the Bayesian Information Criterion (BIC) were then used to confirm the allocations [22].

The multivariable logistic regression models were fit using the *rms* (regression modelling strategies) package [33]. Odds Ratios for continuous variables modelled with restricted cubic splines were estimated comparing the 75th to 25th percentile. The logistic regression model was fit to each imputed dataset and results were combined across the 10 datasets using Rubin’s method which computes imputation-adjusted variances and average betas [30, 34]. Analyses of outcomes with a low frequency of events were conducted using penalized maximum likelihood. The best penalty factor was identified by tracing effective AIC for different penalties [22, 33].

### Subgroup analyses

Subgroup analyses examined the modifying effect of the MTHFR 677C>T genotype and high-risk pregnancy, defined as chronic hypertension, diabetes, history of a PMC, or BMI greater than 35. This was done by including interaction terms with homocysteine and each potential moderator. Subgroup analyses were not conducted for placental abruption and pregnancy loss due to a low number of events.

## Results

**Fig. 1:**
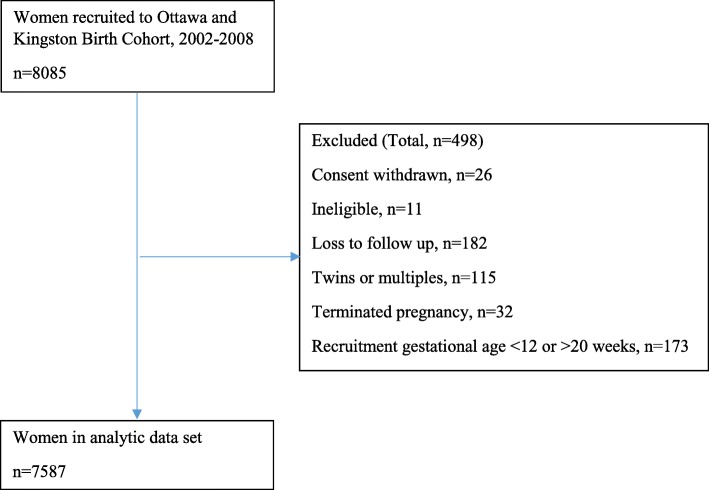
Participant flow diagram for the analytic dataset

Of the 8085 women recruited to the OaK Birth Cohort, 7587 were included in our study (Fig. 1). Descriptive characteristics of participants are presented in Table 1. Maternal education and household income were highly correlated; income was dropped from multivariable analyses because education was more strongly associated with the outcomes. ANOVA plots of partial associations revealed relatively strong associations between homocysteine and the outcomes any placenta-mediated complication (PMC) and SGA (Additional file 2). Nevertheless, AIC and BIC values confirmed that for each outcome the models with homocysteine specified as a simple linear term provided the best fit to the data. The final models included both serum folate and folic acid supplementation because excluding folate did not substantially change effect estimates for folic acid supplementation. We identified two outliers with homocysteine > 20 μmol/L that were included in the final models because exclusion yielded similar results (data not shown).

**Table 1:** Participant characteristics

Variable	Frequency *n* = 7587	
Age		
Mean (SD)	30.3 (5.06)	
Race ^a^ (missing/unknown *N* = 415, 5.5%)		
African	152	(2.12%)
Middle eastern	224	(3.12%)
Asian	422	(5.88%)
Caucasian	6250	(87.1%)
Other	124	(1.73%)
BMI (missing *N* = 136, 1.8%)		
Mean (SD)	24.9 (5.5)	
Range	14.7–61.3	
Participant education (missing N = 7, 0.09%)		
Grade school	153	(2.02%)
High school	962	(12.7%)
College/University not completed	754	(10.0%)
College/University completed	5711	(75.3%)
Paternal/partner education (missing *N* = 95, 1.25%)		
Grade school	137	(1.83%)
High school	1519	(20.3%)
College/University not completed	590	(7.88%)
College/University completed	5246	(70.0%)
Household income (missing *N* = 485, 6.4%)		
< 25 k	415	(5.84%)
25 k - < 50 k	1188	(16.7%)
50 k - < 80 k	2077	(29.2%)
> =80 k	3422	(48.2%)
MTHFR genotype ^b^		
CC (wild type)	1768	(44.1%)
CT (heterozygous)	1760	(43.9%)
TT (mutant)	478	(11.9%)

^a^Race was categorized similar to the U.S. Census, in which the categories are: White, Black or African American, American Indian or Alaska Native, Asian (Far-east and Indian subcontinent), and Hawaiian or Pacific Islander. Participants whose response suggested a Central/South American, Latino, Hispanic, or Aboriginal background were classified into the ‘Other’ category. This follows the U.S. Census Bureau’s classification that: “People who identify their origin as Hispanic, Latino, or Spanish may be of any race” [51]

^b^Measured in a subset of participants (*n* = 4006)

### Effect of homocysteine

Homocysteine concentration was significantly higher in participants with the composite outcome of any PMC, small for gestational age (SGA) infants, and pregnancy loss (Additional file 1: Table A), also demonstrated visually by boxplots of the homocysteine distribution according to pregnancy outcome (Additional file 1: Figure A). In adjusted analyses, higher plasma homocysteine concentration was significantly associated with increased odds of any PMC (Table 2, *p* = 0.0007), SGA (Table 3, *p* = 0.0010), was marginally associated with preeclampsia (*p* = 0.07), and was not associated with placental abruption, and pregnancy loss (Table 3, 0.99, and 0.16). Additionally, homocysteine was associated with severe SGA (<5th percentile) and marginally with severe preeclampsia (delivery < 35 weeks gestation) (Table 3, 0.0012, and 0.0595). A 5 μmol/L change in homocysteine concentration, which is approximately 4 SDs of the homocysteine concentration, was associated with a 63% increased odds of any PMC (Odds Ratio (OR) 1.63, 95% Confidence Interval (CI) 1.23–2.16) and a 76% increased odds of SGA (OR 1.76, 95% CI 1.25–2.46). Serum folate and folic acid supplementation were not associated with the outcomes (Table 2 and Additional file 3: Tables C.1-C.4).

**Table 2:** Multivariable logistic regression analysis of the association between homocysteine and any placenta-mediated complication ^a^ (759 events ^b^), n = 7587

Variable	Odds ratio (95% CI)		*p*-value ^c^
Homocysteine (linear)			0.0007
5 μmol/L increase	1.629	(1.227, 2.161)	
Age (restricted cubic spline, three knots)			0.0031
34 versus 27 years	1.187	(1.063, 1.325)	
Race			0.0002
Caucasian versus others	0.644	(0.509, 0.814)	
Education			0.0056
College/University completed versus less than completed	0.763	(0.630, 0.924)	
Nulliparous			< 0.0001
Yes versus no	1.941	(1.636, 2.303)	
Smoking			< 0.0001
No	Reference		
Second-hand	0705	(0.392, 1.268)	
Med/light smoker (< 10 cigarettes per day)	1.631	(1.228, 2.166)	
Heavy smoker (> 10 cigarettes per day)	1.921	(1.348, 2.737)	
Diabetes			0.0336
Yes versus no	1.687	(1.041, 2.733)	
BMI (restricted cubic spline, four knots)			0.0499
27.3 versus 21.1 kg/m^2^	1.057	(0.883, 1.265)	
Hormonal birth control prior to conception			0.3692
No	Reference		
Oral	0.927	(0.784, 1.096)	
Injection or IUD	0.732	(0.436, 1.227)	
Chronic hypertension			< 0.0001
Yes versus no	2.750	(1.687, 4.483)	
History of PMC (Preeclampsia, placental abruption, IUGR, stillbirth, loss)			0.0110
Yes versus no	1.359	(1.073, 1.722)	
Folic acid supplementation			0.7328
Yes versus no supplementation	0.943	(0.674, 1.320)	
Serum folate (linear)			0.5326
45.1 versus 30.6 nmol/L	1.025	(0.949, 1.106)	
Gestational age at blood work (restricted cubic spline, four knots)			0.0004
13.7 versus 12.4 weeks	0.939	(0.804, 1.095)	

^a^Any placenta-mediated complication- composite of small for gestational age (SGA) <10th percentile, preeclampsia, placental abruption, and pregnancy loss

^b^79 missing outcome values imputed

^c^Wald test of most meaningful hypotheses, pooled across multiple imputation datasets

**Table 3:** Summary of multivariable logistic regression analyses of the association between homocysteine and placenta-mediated complications, *n* = 7587 ^a^

Outcome variable	Odds ratio (95% CI) ^b^		*p*-value ^c^
Any placenta-mediated complication (759 events ^d^)			
Homocysteine (linear)			0.0007
5 μmol/L increase	1.629	(1.227, 2.161)	
SGA (512 events ^d^)			
Homocysteine (linear)			0.0010
5 μmol/L increase	1.756	(1.254, 2.458)	
SGA < 5th percentile (221 events ^d^)			
Homocysteine (linear)			0.0012
5 μmol/L increase	2.022	(1.322, 3.092)	
Preeclampsia (227 events)			
Homocysteine (linear)			0.0736
5 μmol/L increase	1.546	(0.959, 2.491)	
Severe preeclampsia (43 events)			
Homocysteine (linear)			0.0595
5 μmol/L increase	1.762	(0.978, 3.177)	
Placental abruption (68 events)			
Homocysteine (linear)			0.9851
5 μmol/L increase	1.005	(0.590, 1.711)	
Pregnancy loss (85 events)			
Homocysteine (linear)			0.1586
5 μmol/L increase	1.392	(0.879, 2.206)	

^a^Complete results reported in Table 2: any placenta-mediated complication, Additional file 4, and Additional file 3: Table C.1: SGA, Table C.2: Preeclampsia, Table C.3: Placental abruption, and Table C.4: Pregnancy loss

^b^Models adjusted for maternal age, race, education, parity, smoking, diabetes, BMI, hormonal birth control prior to conception, chronic hypertension, history of a placenta mediated complication, folic acid supplementation, serum folate, and gestational age at blood work

^c^Wald test of most meaningful hypotheses, pooled across multiple imputation datasets

^d^79 Missing outcome values imputed

### Subgroup analyses

**Fig. 2:**
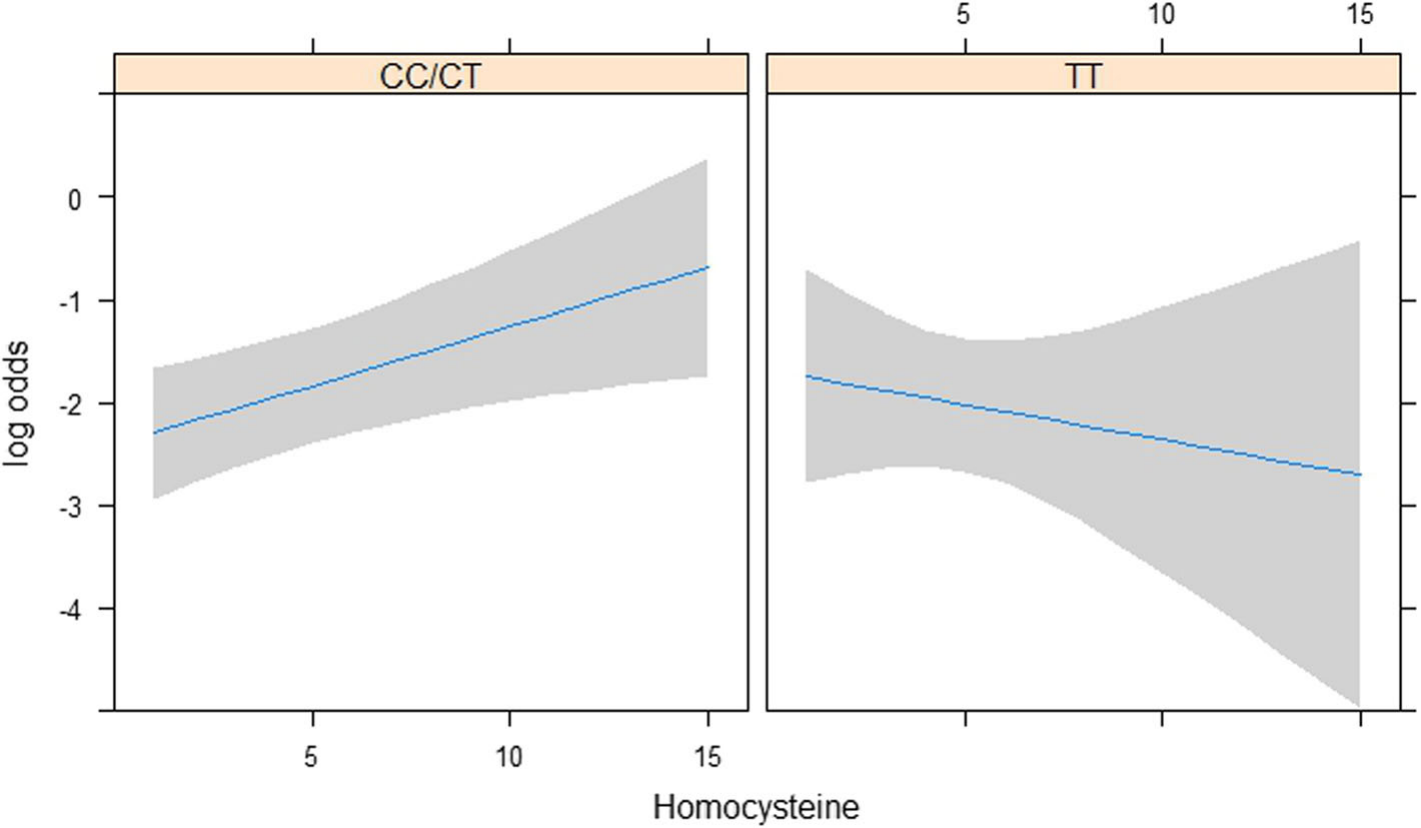
Modelled association between plasma homocysteine (linear) and any placenta-mediated complication, by MTHFR 677C>T genotype CC/CT (wild type and heterozygous) and TT (mutant). Shaded area represents 95% CI

The results of the subgroup analyses are presented in Tables 4 and 5. In the subset of 4006 OaK participants with measured genotype, the interaction between MTHFR 677C>T and homocysteine was not statistically significant for preeclampsia (*p* = 0.84) but there was some evidence of interaction for any PMC (*p* = 0.12) and for SGA (*p* = 0.07) with associations qualitatively different within each subgroup. Associations in the CC/CT subgroups were positive and significant, whereas in TT subgroup associations were negative and not significant, with wide confidence intervals. Figure 2 illustrates these trajectories for the composite outcome with homocysteine. The interaction between high-risk pregnancy and homocysteine was not statistically significant for any PMC and SGA (*p* = 0.27 and *p* = 0.51 respectively), but there was some evidence of a moderating effect for preeclampsia (*p* = 0.14, Table 5). While the interaction effect (and hence, the difference in association between the high and low-risk subgroups) was not statistically significant at the conventional 5% level, the odds ratio was higher and statistically significant in the high-risk group (OR 2.84, 95% CI 1.19 to 6.79 for a 5 μmol/L change in homocysteine concentration) compared to a lower and non-statistically significant OR in the low-risk group (OR 1.31, 95% CI 0.74 to 2.30).

**Table 4:** Multivariable logistic regression analyses examining the moderating effect of MTHFR 677C>T genotype ^a^ on the association between homocysteine and placenta-mediated complications, *n* = 4006

Outcome variable	Odds ratio (95% CI) for 5 μmol/L increase ^b^		*p*-value ^c^
	TT	CC/CT	
Any placenta-mediated complication (395 events ^d^)	0.712 (0.243, 2.083)	1.778 (1.159, 2.729)	0.1172
SGA (277 events ^d^)	0.639 (0.161, 2.536)	2.430 (1.450, 4.073)	0.0714
Preeclampsia (109 events)	1.174 (0.523, 2.633)	1.258 (0.682, 2.322)	0.8439

^a^Homocysteine*MTHFR 677C>T genotype (Factor + higher order factors)

^b^Model adjusted for maternal age, race, education, parity, smoking, diabetes, BMI, hormonal birth control, chronic hypertension, history of PMC, folic acid supplementation, serum folate, gestational age at blood work

^c^Wald test of most meaningful hypotheses, pooled across multiple imputation datasets

^d^40 Missing outcome values imputed

**Table 5:** Multivariable logistic regression analyses examining the moderating effect of high-risk pregnancy ^a^ on the association between homocysteine and placenta-mediated complications, *n* = 7587

Outcome variable	Odds ratio (95% CI) for 5 μmol/L increase ^b^		*p*-value ^c^
	High-risk	Low-risk	
Any placenta-mediated complication (759 events ^d^)	2.368 (1.239, 4.525)	1.595 (1.176, 2.163)	0.2714
SGA (512 events ^d^)	2.474 (1.050, 5.828)	1.821 (1.276, 2.597)	0.5081
Preeclampsia (227 events)	2.839 (1.187, 6.792)	1.308 (0.743, 2.302)	0.1351

^a^Homocysteine*High-risk pregnancy (Factor + higher order factors)

^b^Model adjusted for maternal age, race, education, parity, smoking, hormonal birth control, folic acid supplementation, serum folate, gestational age at blood work

^c^Wald test of most meaningful hypotheses, pooled across multiple imputation datasets

^d^79 Missing outcome values imputed

### Effects of other factors

Different groups of risk factors were associated with SGA and preeclampsia (Tables 3, C.1-C.2). A high school or incomplete post-secondary education and smoking were associated with increased odds for any PMC and SGA. Chronic hypertension, diabetes, and history of experiencing a PMC were associated with increased odds for any PMC and preeclampsia.

## Discussion

### Main findings

We analyzed data from 7587 participants from the Ottawa and Kingston (OaK) Birth cohort. We found that maternal homocysteine concentration in the early to mid-second trimester was associated with increased odds of any placenta-mediated complication (PMC): a composite of small for gestational age (SGA), preeclampsia, placental abruption, and pregnancy loss, and was associated with increased odds of SGA and severe SGA and preeclampsia. In the high-risk subgroup homocysteine was associated with increased odds of preeclampsia.

### Strengths and limitations

To our knowledge, this is the largest cohort study to investigate the association between early to mid-second trimester maternal homocysteine concentration and the risk of placenta-mediated complications. We used multiple imputation to deal with missing values and conducted rigorous multivariable logistic regression analyses designed to explore flexible functional forms of association with homocysteine and other continuous factors, controlling for a wide range of potential confounders.

In most larger studies, homocysteine was dichotomized or grouped to investigate non-linearity and a threshold effect [12, 16]. However, the Hordaland Homocysteine study of several thousand participants suggested that for most conditions including pregnancy complications and adverse pregnancy outcomes, homocysteine exhibits a continuous concentration-response relation [1, 27]. Thus, one of the main strengths of our study is that we accounted for the functional form, i.e., shape of the association, of homocysteine and other continuous variables in relation to the outcomes of interest, which to the best of our knowledge has not been previously reported.

One of the main limitations of our study is that we did not examine the effect of Vitamins B_6_ and B_12_. In a survey of the Canadian population from 2007 to 2009, Vitamin B_12_ was the main determinant of elevated homocysteine concentration in the folate replete [35]. We would expect OaK participants to have adequate Vitamin B_12_ levels because 85% were supplementing with multivitamin or prenatal vitamin supplements, which likely contained Vitamin B_12_. Caffeine or coffee consumption is another homocysteine determinant we did not examine [9], though pregnant women tend to consume less caffeine during pregnancy.

### Interpretation

Other studies have reported an increased risk of SGA associated with elevated homocysteine [12, 36]. In Bergen et al.’s cohort of 5085 participants recruited in the Netherlands from 2002 to 2006, early second trimester homocysteine in the upper versus lower quintile was associated with an increased risk of SGA (<5th centile) [12]. In Dodds et al.’s cohort of 2119 participants recruited in Nova Scotia, Canada from 2002 to 2005, a study time frame similar to our OaK birth cohort, a homocysteine concentration greater than the 90th percentile was not associated with a risk of SGA [13]. The mean homocysteine concentration in the latter study was lower than Bergen et al. [12], but comparable to our participants’ mean homocysteine concentration of 4.8 μmol/L. In another study of 65 SGA cases and 358 controls recruited in a Chinese textile factory from 1996 to 1998, pre-conception homocysteine above the 90th percentile was not associated with an increased risk of SGA [16].These latter two studies dichotomized homocysteine concentration using cut-offs that are not necessarily physiological, which may limit comparability with our findings and those of Bergen et al. [12]

The MTHFR 677C>T genotype qualitatively modified the association of homocysteine with any PMC and SGA. However uncertainty around the negative effect of the TT genotype, likely due to fewer participants the TT subgroup, makes it difficult to interpret the modifying effect. Higher folate intake and status are known to mitigate but not eliminate the effect of this polymorphism on homocysteine concentration [19, 37, 38]. Although approximately 95% of our sample was consuming a folic acid supplement and white flour and other cereal products in Canada have been fortified with folic acid since the late 1990s, homocysteine concentration was associated with the MTHFR genotype in the OaK cohort. Given the variations in clinical testing for MTHFR genotype in current obstetric practice [39], our study findings suggest testing may be warranted, particularly in high-risk subgroups, for example, those who exhibit comorbidities or a history of complications.

We found that elevated homocysteine was marginally associated with an increased risk of preeclampsia, and that the association was significant in the high-risk subgroup. Others have found no association [12, 14]; for example, Kahn et al.’s nested case-control study with 113 preeclampsia cases and 443 controls recruited in Montreal from 1999 to 2004 [14]. Studies of repeated homocysteine measurement during pregnancy have reported increasing homocysteine concentration during the course of preeclampsia [40]. In a longitudinal analysis of 252 women of whom 49 developed preeclampsia, homocysteine increased in the preeclampsia group independent of B-vitamin (B_6_, B_12_, and folate) and obesity status, while concentrations in the uncomplicated group remained steady [41]. Therefore early homocysteine measurement, as in our study, may pre-date onset of disease and changes in homocysteine. Our finding of a stronger association of homocysteine in severe preeclampsia, marginally significant with fewer outcome events, also suggests a role of homocysteine earlier in pregnancy when severity is increased.

Some studies have, however, reported an increased risk of preeclampsia associated with higher homocysteine concentrations [13, 42–45]. Many of these studies were conducted during a time period before routine peri-conceptional folic acid supplementation and/or mandatory folic acid fortification (i.e., the early to mid-1990s) [42–44], or were conducted in countries without mandatory folic acid fortification [45–47]. This suggests that where folate intake is lower, homocysteine levels would tend to be higher and therefore homocysteine might play a greater role, earlier on, in the development of preeclampsia.

We report the association of the range of potential confounders with the composite and individual outcomes. Our results, although exploratory in this regard, demonstrated a greater role of diabetes and chronic hypertension in the development of preeclampsia compared to SGA. In a clinical opinion paper, Ness and Sibai [48] hypothesized that the maternal syndrome observed in preeclampsia develops in the presence of abnormal placentation that interacts with maternal metabolic syndrome, and that fetal growth restriction develops in the absence of metabolic syndrome. Our findings of increased odds of preeclampsia in the high-risk subgroup also lend support to this hypothesized role of developing endothelial dysfunction in preeclampsia.

In our study elevated homocysteine concentration was not associated with an increased risk of placental abruption and pregnancy loss, but a greater number of events would be necessary to confirm an association. Some studies have found no association of homocysteine with early pregnancy loss [15, 17, 49]. However, one-time early pregnancy losses are characteristically different from recurrent early pregnancy losses and losses throughout pregnancy [10].

## Conclusions

In summary, our results support an independent effect of early to mid-pregnancy elevated homocysteine on placenta-mediated pregnancy complications. Our findings are comparable with similar large studies; high-risk pregnancy and potentially the MTHFR 677C>T genotype may contribute to some of the observed differences between studies. As with ongoing investigations into the role of homocysteine in cardiovascular disease, large Mendelian randomization studies could further confirm the etiological role of homocysteine in placenta-mediated pregnancy complications [50].

## Additional files

Additional file 1: Homocysteine distribution in the entire sample and by outcome. (DOCX 145 kb)

Additional file 2: ANOVA plots of partial associations from saturated model for each outcome. (DOCX 433 kb)

Additional file 3: Complete tables of results for the multivariable logistic regression analyses. (DOCX 27 kb)

Additional file 4: Modelled associations of restricted cubic spline functions. (DOCX 1090 kb)

## Abbreviations

AIC: Akaike’s information criterion
BIC: Bayesian information criterion
BMI: Body mass index
IUGR: Intrauterine growth restriction
MTHFR: Methylenetetrahydrofolate reductase
OaK: Ottawa and Kingston
PMC: Placenta-mediated complication
SGA: Small for gestational age
