# Supplementary material for: The role of maternal homocysteine concentration in placenta-mediated complications: findings from the Ottawa and Kingston birth cohort
Source: BMC Pregnancy Childbirth. 2019 Feb 19;19:75. doi: 10.1186/s12884-019-2219-5 (PMC6381683; doi:10.1186/s12884-019-2219-5)
Supplement: Supplementary file 1 — Homocysteine distribution in the entire sample and by outcome. (DOCX 145 kb) [file 12884_2019_2219_MOESM1_ESM.docx]

**Additional file 1**

**Homocysteine distribution**

**Table A: Homocysteine concentration according to placenta-mediated complication**

| **Homocysteine µmol/L** | **Outcome** | | **P-value** ^a^ |
| --- | --- | --- | --- |
|  | **Yes** | **No** |  |
| **Entire cohort** | n=7500 ^b^ | |  |
| Mean (SD) | 4.83 (1.27) | |  |
| Median (Q1-Q3) | 5 (4-5) | |  |
| Range | 1-34 | |  |
| **Any placenta-mediated complication** | n=745 | n=6676 | 0.0001 |
| Mean (SD) | 5.03 (1.51) | 4.81 (1.25) |  |
| Median (Q1-Q3) | 5 (4-6) | 5 (4-5) |  |
| Range | 2-24 | 1-34 |  |
| **Preeclampsia** | n=223 | n=7277 | 0.0880 |
| Mean (SD) | 4.99 (1.43) | 4.82 (1.27) |  |
| Median (Q1-Q3) | 5 (4-6) | 5 (4-5) |  |
| Range | 2-14 | 1-34 |  |
| **Small for gestational age** | n=502 | n=6919 | <0.0001 |
| Mean (SD) | 5.11 (1.63) | 4.81 (1.25) |  |
| Median (Q1-Q3) | 5 (4-6) | 5 (4-5) |  |
| Range | 2-24 | 1-34 |  |
| **Placental abruption** | n=65 | n=7435 | 0.9238 |
| Mean (SD) | 4.82 (1.20) | 4.83 (1.28) |  |
| Median (Q1-Q3) | 5 (4-5) | 5 (4-5) |  |
| Range | 3-11 | 1-34 |  |
| **Pregnancy loss** | n=85 | n=7415 | 0.0332 |
| Mean (SD) | 5.19 (1.54) | 4.83 (1.27) |  |
| Median (Q1-Q3) | 5 (4-6) | 5 (4-5) |  |
| Range | 3-14 | 1-34 |  |

^a^ Welch two sample t test

^b^ 87 missing homocysteine measurement

**
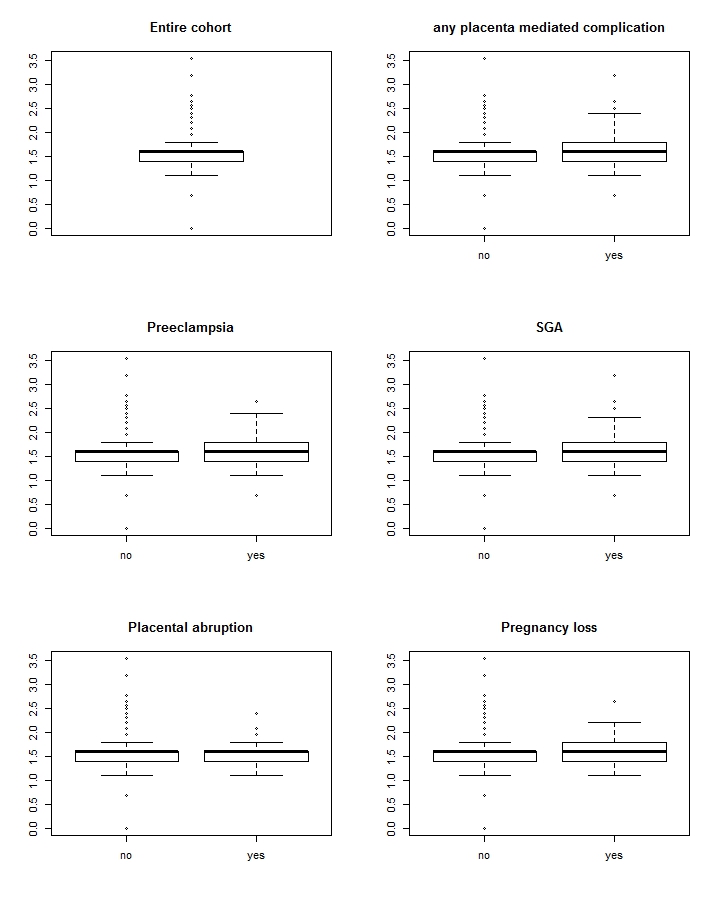
**

**Figure A:** Boxplot of log-transformed plasma homocysteine concentration (µmol/L) in entire cohort and according to pregnancy outcome
