# Supplementary material for: The role of maternal homocysteine concentration in placenta-mediated complications: findings from the Ottawa and Kingston birth cohort
Source: BMC Pregnancy Childbirth. 2019 Feb 19;19:75. doi: 10.1186/s12884-019-2219-5 (PMC6381683; doi:10.1186/s12884-019-2219-5)
Supplement: Supplementary file 2 — ANOVA plots of partial associations from saturated model for each outcome. (DOCX 433 kb) [file 12884_2019_2219_MOESM2_ESM.docx]

**Additional file 2**

**ANOVA plots of partial associations**

ANOVA plots of partial associations, Chi-squared minus degrees of freedom, were generated to identify strong and weak partial associations. An ANOVA plot demonstrates the predictive ability of each variable while not displaying tests of significance. For each predictor, the strength of association is used as a guide to allocate degrees of freedom for the final model: strong associations are modeled with greater complexity than weak associations.


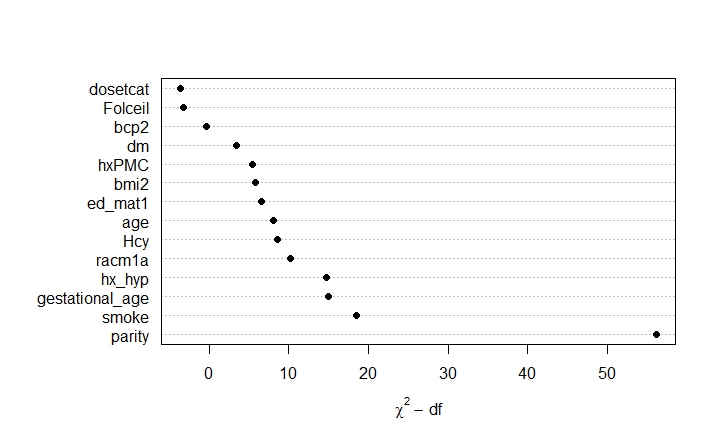


**Figure B.1**: ANOVA plot of partial associations from saturated model for any placenta-mediated complication (Table 3). Hcy: homocysteine.


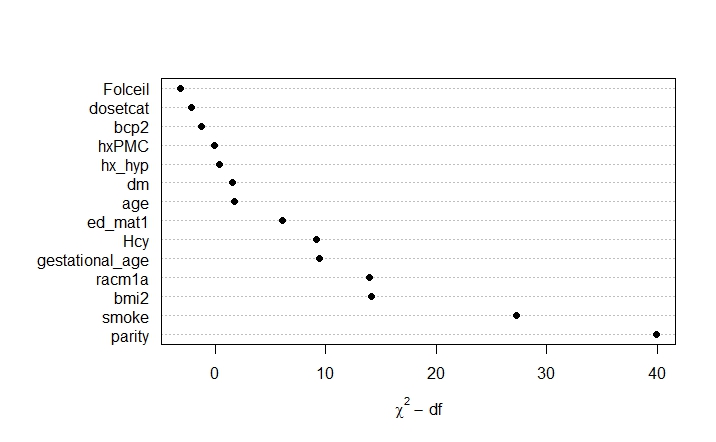


**Figure B.2**: ANOVA plot of partial associations from saturated model for small for gestational age (Table C.1). Hcy: homocysteine.


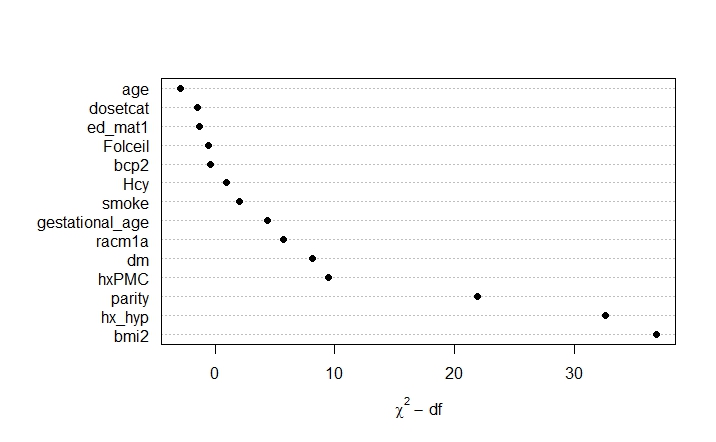


**Figure B.3**: ANOVA plot of partial associations from saturated model for preeclampsia (Table C.2). Hcy: homocysteine.

.


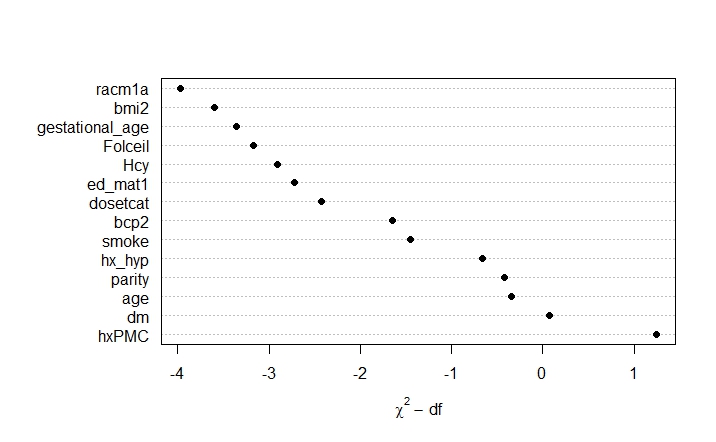


**Figure B.4**: ANOVA plot of partial associations from saturated model for placental abruption (Table C.3). Hcy: homocysteine.

**
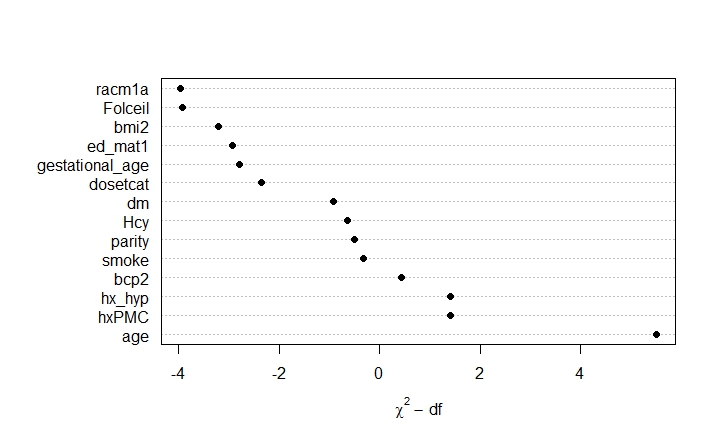
**

**Figure B.5**: ANOVA plot of partial associations from saturated model for pregnancy loss (Table C.4). Hcy: homocysteine.
