# Supplementary material for: The role of maternal homocysteine concentration in placenta-mediated complications: findings from the Ottawa and Kingston birth cohort
Source: BMC Pregnancy Childbirth. 2019 Feb 19;19:75. doi: 10.1186/s12884-019-2219-5 (PMC6381683; doi:10.1186/s12884-019-2219-5)
Supplement: Supplementary file 3 — Complete tables of results for the multivariable logistic regression analyses. (DOCX 27 kb) [file 12884_2019_2219_MOESM3_ESM.docx]

**Additional file 3**

**Complete results for multivariable logistic regression analyses**

**Table C.1: Multivariable logistic regression analysis of the association between homocysteine and SGA (512 events** ^a^**), n=7587**

| **Variable** | **Odds ratio (95% CI)** | | **p-value** ^b^ |
| --- | --- | --- | --- |
| **Homocysteine** (linear) |  |  | 0.0010 |
| 5 µmol/L increase | 1.756 | (1.254, 2.458) |  |
| **Age** (linear) |  |  | 0.0538 |
| 34 versus 27 years | 1.151 | (0.998, 1.328) |  |
| **Race** |  |  | 0.0001 |
| Caucasian versus others | 0.578 | (0.437, 0.766) |  |
| **Education** |  |  | 0.0038 |
| College/University completed versus less than completed | 0.714 | (0.568, 0.897) |  |
| **Nulliparous** |  |  | <0.0001 |
| Yes versus no | 2.063 | (1.662, 2.560) |  |
| **Smoking** |  |  | <0.0001 |
| No | Reference |  |  |
| Second-hand | 0.845 | (0.422, 1.694) |  |
| Med/light smoker (<10 cigarettes per day) | 2.205 | (1.600, 3.038) |  |
| Heavy smoker (>10 cigarettes per day) | 2.359 | (1.554, 3.580) |  |
| **Diabetes** |  |  | 0.1085 |
| Yes versus no | 0.432 | (0.155, 1.204) |  |
| **BMI** (restricted cubic spline, 5 knots) |  |  | 0.0004 |
| 27.3 versus 21.1 kg/m^2^ | 0.803 | (0.625, 1.031) |  |
| **Hormonal birth control prior to conception** |  |  | 0.6512 |
| No | Reference |  |  |
| Oral | 0.956 | (0.780, 1.173) |  |
| Injection or IUD | 0.752 | (0.395, 1.431) |  |
| **Chronic hypertension** |  |  | 0.1981 |
| Yes versus no | 1.610 | (0.780, 3.323) |  |
| **History of PMC** (Preeclampsia, placental abruption, IUGR, stillbirth, loss) |  |  | 0.3037 |
| Yes versus no | 1.179 | (0.861, 1.614) |  |
| **Folic acid supplementation** |  |  | 0.7468 |
| Yes versus no supplementation | 1.074 | (0.697, 1.654) |  |
| **Serum folate** (linear) |  |  | 0.4243 |
| 45.1 versus 30.6 nmol/L | 1.039 | (0.946, 1.140) |  |
| **Gestational age at blood work** (restricted cubic spline, three knots) |  |  | 0.0612 |
| 13.7 versus 12.4 weeks | 0.816 | (0.670, 0.994) |  |

^a^ Additional 79 missing values imputed

^b^ Wald test of most meaningful hypotheses, pooled across multiple imputation datasets

**Table C.2: Multivariable logistic regression analysis of the association between homocysteine and preeclampsia (227 events), n=7587**

| **Variable** | **Odds ratio (95% CI)** | | **p-value** ^a^ |
| --- | --- | --- | --- |
| **Homocysteine** (linear) |  |  | 0.0736 |
| 5 µmol/L increase | 1.546 | (0.959, 2.491) |  |
| **Age** (linear) |  |  | 0.8614 |
| 34 versus 27 years | 1.019 | (0.829, 1.252) |  |
| **Race** |  |  | 0.0362 |
| Caucasian versus others | 0.627 | (0.405, 0.970) |  |
| **Education** |  |  | 0.2625 |
| College/University completed versus less than completed | 0.826 | (0.591, 1.154) |  |
| **Nulliparous** |  |  | <0.0001 |
| Yes versus no | 2.353 | (1.662, 3.332) |  |
| **Smoking** |  |  | 0.1693 |
| No | Reference |  |  |
| Second-hand | 0.298 | (0.072, 1.244) |  |
| Med/light smoker (<10 cigarettes per day) | 0.605 | (0.300, 1.220) |  |
| Heavy smoker (>10 cigarettes per day) | 1.175 | (0.585, 2.358) |  |
| **Diabetes** |  |  | 0.0012 |
| Yes versus no | 2.809 | (1.506, 5.239) |  |
| **BMI** (restricted cubic spline, four knots) |  |  | <0.0001 |
| 27.3 versus 21.1 kg/m^2^ | 2.428 | (1.757, 3.356) |  |
| **Hormonal birth control prior to conception** |  |  | 0.3977 |
| No | Reference |  |  |
| Oral | 1.114 | (0.826, 1.504) |  |
| Injection or IUD | 0.515 | (0.157, 1.695) |  |
| **Chronic hypertension** |  |  | <0.0001 |
| Yes versus no | 5.824 | (3.302, 10.27) |  |
| **History of PMC** (Preeclampsia, placental abruption, IUGR, stillbirth, loss) |  |  | 0.0007 |
| Yes versus no | 1.981 | (1.333, 2.943) |  |
| **Folic acid supplementation** |  |  | 0.2458 |
| Yes versus no supplementation | 0.712 | (0.401, 1.264) |  |
| **Serum folate** (linear) |  |  | 0.5877 |
| 45.1 versus 30.6 nmol/L | 1.041 | (0.900, 1.203) |  |
| **Gestational age at blood work** (linear) |  |  | 0.1720 |
| 13.7 versus 12.4 weeks | 1.057 | (0.976, 1.144) |  |

^a^ Wald test of most meaningful hypotheses, pooled across multiple imputation datasets

**Table C.3: Multivariable logistic regression analysis of the association between homocysteine and placental abruption (68 events), n=7587**

| **Variable** | **Odds ratio (95% CI)** | | **p-value** ^a^ |
| --- | --- | --- | --- |
| **Homocysteine** (linear) |  |  | 0.9851 |
| 5 µmol/L increase | 1.005 | (0.590, 1.711) |  |
| **Age** (linear) |  |  | 0.2044 |
| 34 versus 27 years | 1.130 | (0.935, 1.366) |  |
| **Race** |  |  | 0.9133 |
| Caucasian versus others | 1.026 | (0.642, 1.640) |  |
| **Education** |  |  | 0.7271 |
| College/University completed versus less than completed | 1.059 | (0.769, 1.457) |  |
| **Nulliparous** |  |  | 0.3963 |
| Yes versus no | 0.887 | (0.673, 1.170) |  |
| **Smoking** |  |  | 0.6952 |
| No | Reference |  |  |
| Second-hand | 1.049 | (0.860, 1.280) |  |
| Med/light smoker (<10 cigarettes per day) | 1.090 | (0.575, 2.067) |  |
| Heavy smoker (>10 cigarettes per day) | 1.633 | (0.732, 3.645) |  |
| **Diabetes** |  |  | 0.2926 |
| Yes versus no | 1.704 | (0.632, 4.594) |  |
| **BMI** (restricted cubic spline, four knots) |  |  | 0.8112 |
| 27.3 versus 21.1 kg/m^2^ | 0.982 | (0.842, 1.144) |  |
| **Hormonal birth control prior to conception** |  |  | 0.8030 |
| No | Reference |  |  |
| Oral | 0.931 | (0.752, 1.151) |  |
| Injection or IUD | 0.968 | (0.773, 1.213) |  |
| **Chronic hypertension** |  |  | 0.5191 |
| Yes versus no | 1.451 | (0.468, 4.496) |  |
| **History of PMC** (Preeclampsia, placental abruption, IUGR, stillbirth, loss) |  |  | 0.1148 |
| Yes versus no | 1.401 | (0.921, 2.129) | 0.7765 |
| **Folic acid supplementation** |  |  |  |
| Yes versus no supplementation | 1.092 | (0.595, 2.002) |  |
| **Serum folate** (linear) |  |  | 0.4896 |
| 45.1 versus 30.6 nmol/L | 0.954 | (0.835, 1.090) |  |
| **Gestational age at blood work** (linear) |  |  | 0.5598 |
| 13.7 versus 12.4 weeks | 1.025 | (0.944, 1.112) |  |

^a^ Wald test of most meaningful hypotheses, pooled across multiple imputation datasets

**Table C.4: Multivariable logistic regression analysis of the association between homocysteine and pregnancy loss (85 events), n=7587**

| **Variable** | **Odds ratio (95% CI)** | | **p-value** ^a^ |
| --- | --- | --- | --- |
| **Homocysteine** (linear) |  |  | 0.1586 |
| 5 µmol/L increase | 1.392 | (0.879, 2.206) |  |
| **Age** (linear) |  |  | 0.0456 |
| 34 versus 27 years | 1.265 | (1.021, 1.567) |  |
| **Race** |  |  | 0.6982 |
| Caucasian versus others | 1.099 | (0.681, 1.775) |  |
| **Education** |  |  | 0.7958 |
| College/University completed versus less than completed | 0.958 | (0.691, 1.327) |  |
| **Nulliparous** |  |  | 0.4722 |
| Yes versus no | 0.900 | (0.676, 1.199) |  |
| **Smoking** |  |  | 0.4109 |
| No | Reference |  |  |
| Second-hand | 1.110 | (0.901, 1.367) |  |
| Med/light smoker (<10 cigarettes per day) | 1.464 | (0.800, 2.677) |  |
| Heavy smoker (>10 cigarettes per day) | 1.716 | (0.804, 3.664) |  |
| **Diabetes** |  |  | 0.7976 |
| Yes versus no | 1.149 | (0.397, 3.321) |  |
| **BMI** (restricted cubic spline, four knots) |  |  | 0.4262 |
| 27.3 versus 21.1 kg/m^2^ | 1.064 | (0.913, 1.241) |  |
| **Hormonal birth control prior to conception** |  |  | 0.2841 |
| No | Reference |  |  |
| Oral | 0.832 | (0.663, 1.045) |  |
| Injection or IUD | 0.922 | (0.721, 1.178) |  |
| **Chronic hypertension** |  |  | 0.1096 |
| Yes versus no | 2.229 | (0.835, 5.950) |  |
| **History of PMC** (Preeclampsia, placental abruption, IUGR, stillbirth, loss) |  |  | 0.1192 |
| Yes versus no | 1.393 | (0.918, 2.113) |  |
| **Folic acid supplementation** |  |  | 0.7994 |
| Yes versus no supplementation | 0.925 | (0.508, 1.686) |  |
| **Serum folate** (linear) |  |  | 0.8072 |
| 45.3 versus 30.7 nmol/L | 0.983 | (0.860, 1.125) |  |
| **Gestational age at blood work** (linear) |  |  | 0.3440 |
| 13.7 versus 12.4 weeks | 0.959 | (0.879, 1.046) |  |

^a^ Wald test of most meaningful hypotheses, pooled across multiple imputation datasets
