# Supplementary material for: The role of maternal homocysteine concentration in placenta-mediated complications: findings from the Ottawa and Kingston birth cohort
Source: BMC Pregnancy Childbirth. 2019 Feb 19;19:75. doi: 10.1186/s12884-019-2219-5 (PMC6381683; doi:10.1186/s12884-019-2219-5)
Supplement: Supplementary file 4 — Modelled associations of restricted cubic spline functions. (DOCX 1090 kb) [file 12884_2019_2219_MOESM4_ESM.docx]

**Additional file 4**

**Modelled associations of restricted cubic spline functions**

**
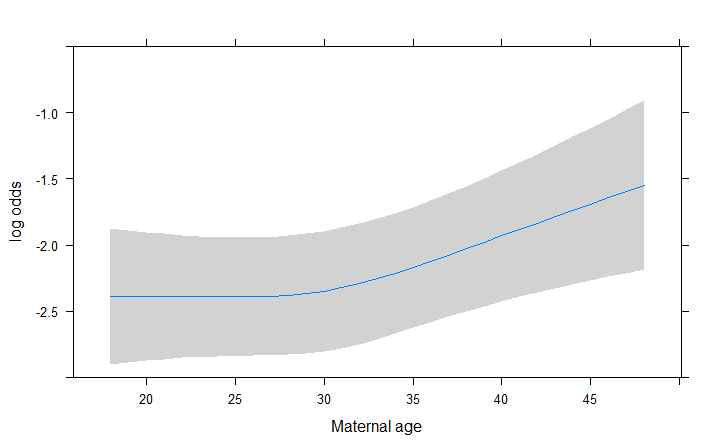
**

**Figure D.1:** Modelled association between maternal age (years) and any placenta-mediated complication, presented in Table 2. Restricted cubic spline with three knots at 24, 30, and 37. Shaded area represents 95% CI.

**
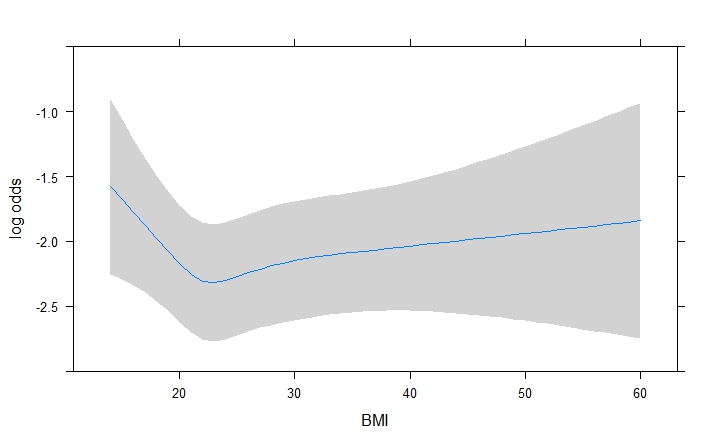
**

**Figure D.2:** Modelled association between BMI (kg/m^2^) and any placenta-mediated complication, presented in Table 2. Restricted cubic spline with four knots at 19, 22, 25, and 36. Shaded area represents 95% CI.

**
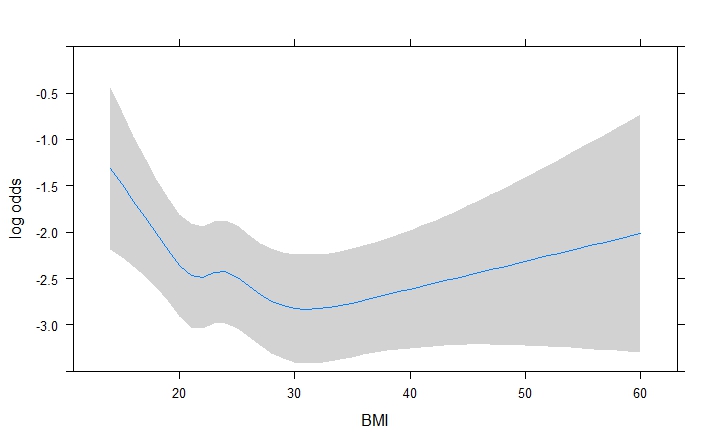
**

**Figure D.3:** Modelled association between BMI (kg/m^2^) and SGA, presented in Table C.1. Restricted cubic spline with five knots at 19, 21, 23, 27, and 36. Shaded area represents 95% CI.

**
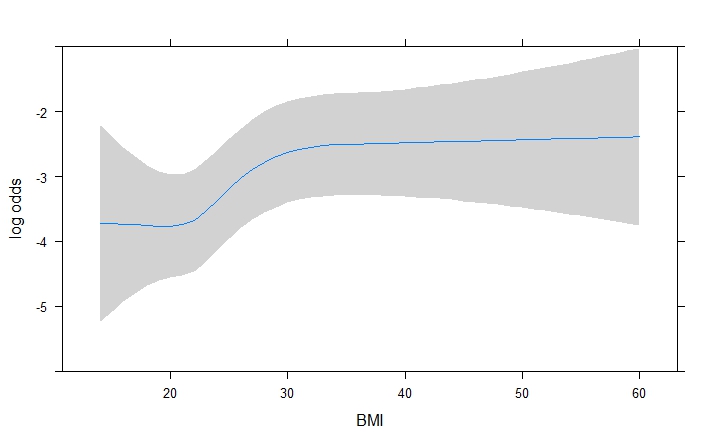
**

**Figure D.4:** Modelled association between BMI (kg/m^2^) and preeclampsia, presented in Table C.2. Restricted cubic spline with four knots at 19, 22, 25, and 36. Shaded area represents 95% CI.
